# Genomic analyses reveal a low-temperature adapted clade in *Halorubrum*, a widespread haloarchaeon across global hypersaline environments

**DOI:** 10.1186/s12864-023-09597-7

**Published:** 2023-08-31

**Authors:** Liangzhong Chen, Tao Hong, Zirui Wu, Weizhi Song, Shaoxing X. Chen, Yongqin Liu, Liang Shen

**Affiliations:** 1 College of Life Sciences, Anhui Normal University, Wuhu, 241000 China; 2 Anhui Provincial Key Laboratory of Conservation and Exploitation of Biological Resources, Anhui Normal University, Wuhu, 241000 China; 3 Anhui Provincial Key Laboratory of Molecular Enzymology and Mechanism of Major Diseases, and Auhui Provincial Engineering Research Centre for Molecular Detection and Diagnostics, Anhui Normal University, Wuhu, 241000 China; 4 Centre for Marine Bio-Innovation, University of New South Wales, Sydney, NSW 2052 Australia; 5 Present Address: Center for the Pan-third Pole Environment, Lanzhou University, Lanzhou, 730000 China; 6 State Key Laboratory of Tibetan Plateau Earth System Science, Environment and Resources (TPESER), Institute of Tibetan Plateau Research, Chinese Academy of Sciences, 100085 Beijing, China

**Keywords:** Genomics, Cold adaptation, Polar and deep Earth environments, Microbial adaptation, Hypersaline environments

## Abstract

**Background:**

Cold-adapted archaea have diverse ecological roles in a wide range of low-temperature environments. Improving our knowledge of the genomic features that enable psychrophiles to grow in cold environments helps us to understand their adaptive responses. However, samples from typical cold regions such as the remote Arctic and Antarctic are rare, and the limited number of high-quality genomes available leaves us with little data on genomic traits that are statistically associated with cold environmental conditions.

**Results:**

In this study, we examined the haloarchaeal genus *Halorubrum* and defined a new clade that represents six isolates from polar and deep earth environments (‘PD group’ hereafter). The genomic G + C content and amino acid composition of this group distinguishes it from other *Halorubrum* and the trends are consistent with the established genomic optimization of psychrophiles. The cold adaptation of the PD group was further supported by observations of increased flexibility of proteins encoded across the genome and the findings of a growth test.

**Conclusions:**

The PD group *Halorubrum* exhibited denser genome packing, which confers higher metabolic potential with constant genome size, relative to the reference group, resulting in significant differences in carbon, nitrogen and sulfur metabolic patterns. The most marked feature was the enrichment of genes involved in sulfur cycling, especially the production of sulfite from organic sulfur-containing compounds. Our study provides an updated view of the genomic traits and metabolic potential of *Halorubrum* and expands the range of sources of cold-adapted haloarchaea.

**Supplementary Information:**

The online version contains supplementary material available at 10.1186/s12864-023-09597-7.

## Background

Habitats suitable for low-temperature adapted microorganisms represent a large proportion of the Earth’s biosphere as over 70% of the Earth’s biosphere has a temperature < 5 ℃ [1–3]. In such habitats, archaea are prevalent and are represented by a diverse array of taxa, which play critical roles in global biogeochemical cycles [2, 4]. The specific environmental conditions of low-temperature habitats also represent a treasure trove for the discovery of new adaptations and evolutionary mechanisms [5]. Given their importance, a series of studies have been conducted to uncover the ecologically significant genomic features of these cold-adapted microbes [2, 6–8].

One of the best-studied psychrophilic archaeon is *Methanococcoides burtonii*, a methylotrophic methanogen isolated from Ace Lake, Antarctica [9]. Comparative genomics has revealed that the cold-adapted archaea were characterized by higher Gln (glutamine) and Thr (threonine) content and lower Leu (leucine) content, as well as high genome plasticity, which induced the acquisition of adaptive genes from Proteobacteria [9, 10]. Proteomic analyses have indicated that *Halorubrum lacusprofundi* responded to low-temperature stressors with a number of synergistic changes, including higher abundance of proteins associated the formation of polyhydroxyalkanoate-like granules and the synthesis of high levels of Hsp20 chaperones [11]. Genomic and proteomic analysis of psychrophilic bacteria has also revealed amino acid composition bias and the presence of specific genes in response to cold temperatures. For example, in *Pseudoalteromonas haloplanktis*, a bias toward Asn (asparagine) has been observed; dioxygen scavenging genes have been enriched while whole pathways producing reactive oxygen species have been lost [12]. The optimization of genome-wide amino acid composition and the presence of specific genes have been observed in psychrophilic microbes such as *Colwellia psychrerythraea* [13], *Psychromonas ingrahamii* [14], *Psychrobacter arcticus* [15] and *Planococcus halocryophilus* [16]. These studies have established a basic understanding of the genomic and proteomic characteristics of the adaptation of microorganisms to cold environments [6, 8, 17].

However, such studies have been limited by the difficulty of collecting samples from remote polar and alpine regions (where the majority of psychrophilic microbes originate) and the high cost of sequencing in the early years, during which the sequencing of a new genome was a feat in itself. Thus, single genomes from taxa of interest have been used to identify genomic features, and these genomes have been compared to genetically distant counterparts (e.g. comparisons between Deltaproteobacteria *Desulfotalea psychrophila* and Gammaproteobacteria *Pseudomonas putida*; and Halobacteriota *Methanococcoides burtonii* and Methanobacteriota *Methanocaldococcus jannaschii*) [9, 18]. Thus, there is still a knowledge gap in the literature, and the identification of genomic traits that are statistically associated with cold-environment conditions based on multiple genomes is required, with the exclusion, as much as possible, of interference caused by genetic distance (i.e. phylogenetic noise) [19, 20]. This situation might be even more pronounced in Archaea, for which high-quality non-redundant microbial genomic data from polar and alpine regions are relatively rare and insufficient [21, 22].

The haloarchaeal genus *Halorubrum* (Halobacteriota; Halobacteria; Halobacteriales; Haloferacaceae) was established by transferring four species from the genus *Halobacterium* in 1995 [23]. In addition to the psychrophilic members of the genus that are abundant in Antarctica’s Deep Lake, *Halorubrum* strains have been isolated and detected in other saline and cold environments globally (e.g. deep salt mines, saline soils, solar salts and Canadian high Arctic permafrost) [11, 24–26]. *Halorubrum* is one of the largest genera (in terms of diversity) of haloarchaea, and more than 40 valid species have been recorded in the literature (https://lpsn.dsmz.de/text/introduction, accessed in Jan. 2022). Importantly, many *Halorubrum* strains have been isolated from deep subterranean salt mines [25]; such mines usually have a relatively low and constant temperature relative to solar salterns, another main habitat of *Halorubrum* [27, 28]. These observations suggest that cold-adapted *Halorubrum* species may not be exclusive to Antarctic lakes.

In this study, we analysed 70 high-quality non-redundant *Halorubrum* genomes derived from diverse hypersaline environments, including Antarctica’s Deep Lake and subterranean salt mines. We first present an updated summary of the general genomic features of *Halorubrum*. After identifying a clade characterized by their adaptation to cold environments, genomic traits that are statistically associated with cold adaptation are identified. Our findings illustrated the important role of *Halorubrum* in driving biogeochemical cycling within cold environments; and expanded the sources of cold-adapted haloarchaea to deep earth environments.

## Results

### Phylogeny of *Halorubrum*

**Fig. 1:**
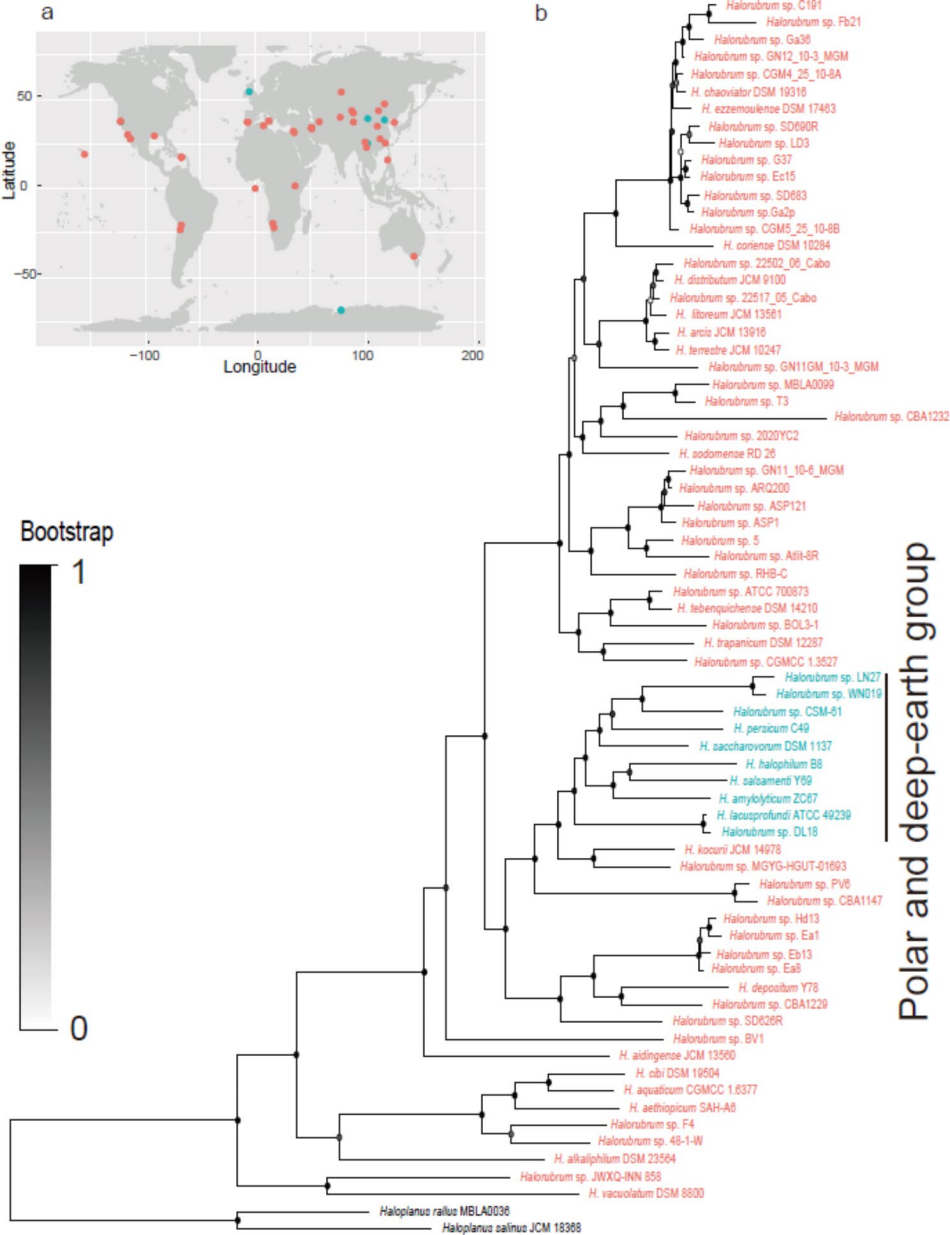
(**a**) Geographical locations of the *Halorubrum* isolates, and (**b**) *Halorubrum* phylogeny reconstructed by PhyloPhlAn 3.0 based on the 400 most universal markers using maximum likelihood algorithm. The central clade highlighted in blue starts with *Halorubrum* sp. DL18, which was isolated from Antarctica’s Deep Lake, and ends with *Halorubrum* sp. LN27, which was isolated from a deep salt column (350 m); this clade represents the polar and deep-earth group (in blue). The bar labelled ‘0.2’ indicates accumulated changes per amino acid. Dots at nodes indicate bootstrap proportions for the tree. The phylogenomic tree was sorted by increasing node order using FigTree, as described in the ‘Materials and methods’ section

For phylogenomic clustering, *Haloplanus rallus* MBLA0036 (NZ_CP034345) and *Haloplanus salinus* JCM 18,368 (NZ_QPHM01000001) were chosen as the outgroup, as they are among the close relatives of *Halorubrum* [28] – species that are closely related to the in-group are more suitable for phylogenetic reconstruction than distantly related species [29]. *Halorubrum* spp. have been isolated from various saline environments globally, including Antarctica’s Deep Lake and salt mines hundreds of meters underground (Fig. 1a, Table S1). A clade in the middle of the tree harboured a higher percentage of isolates derived from deep-earth and Antarctic environments than the upper and lower clades (about 60% in the middle clade vs. < 3% in the upper clades and < 5% in the lower clades, Fig. 1b, please note that the tree has been sorted in increasing node order). This implies that the middle clade may represent a specific ecotype that is well adapted to the polar and deep-earth environments. The genomes belonging to this clade are referred to as the polar and deep-earth group (‘PD group’ hereafter). To investigate the genomic features shared by this group, we performed comparative genomic analysis by setting the remaining genomes as a control (i.e. reference group).

### Low-temperature growth capacity of the PD group *Halorubrum*

The low-temperature growth pattern of the Antarctic isolates has already been well characterized [11]. To further evaluate the growth temperature response of the PD group *Halorubrum*, representative isolates from the PD group (*Halorubrum* sp. LN27) and the reference group (*Halorubrum* sp. T3) were grown at 4 °C on solid agar medium for 30 days. The isolate *Halorubrum* LN27 from the PD group exhibited a clearly enhanced rate of growth at 4 °C compared to *Halorubrum* T3 from the reference group (Fig. S1).

### Pangenome and core genome of *Halorubrum*

**Fig. 2:**
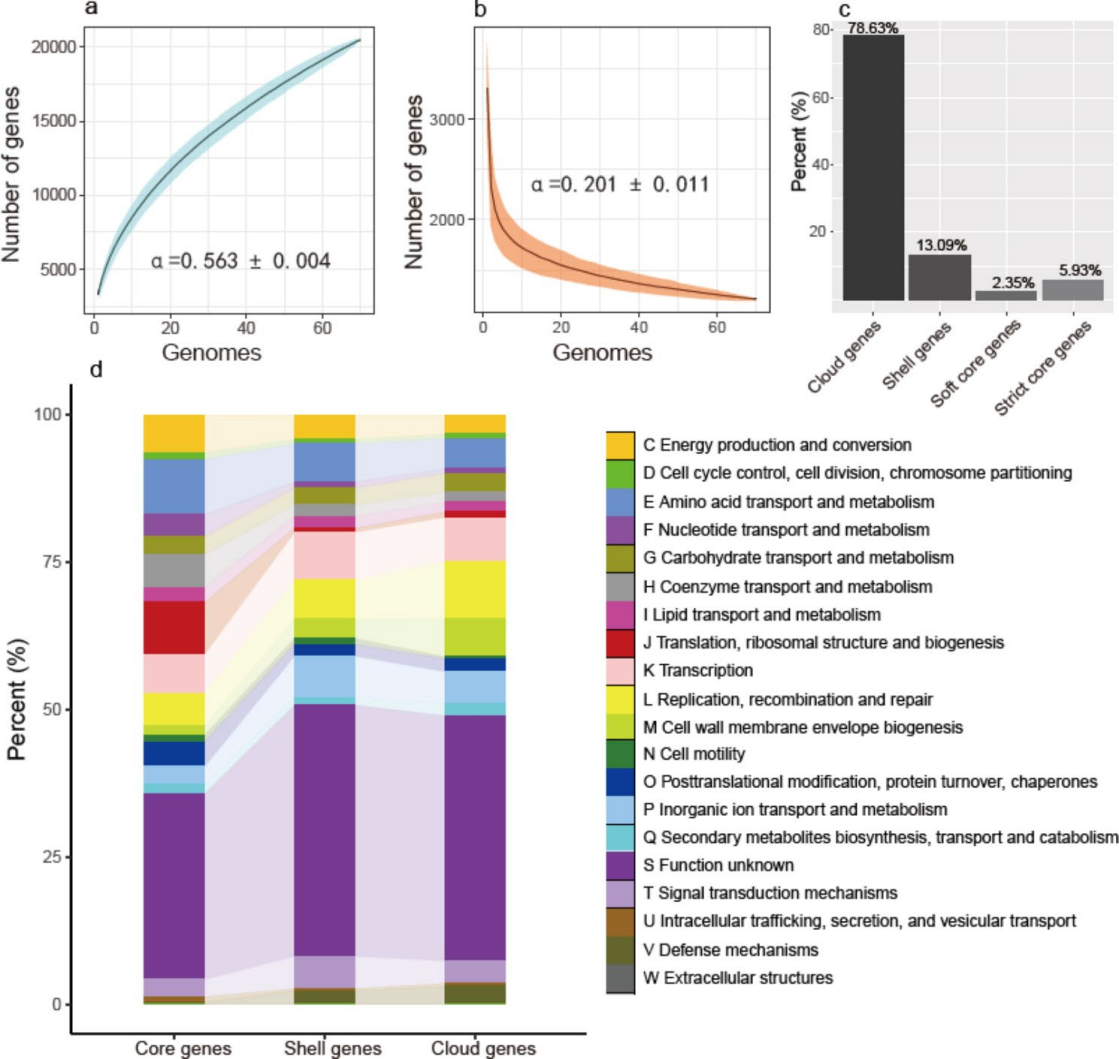
Rarefaction curves of pan and core gene numbers and summary statistics for the pangenome of *Halorubrum*. (**a**) Rarefaction curve for the accumulation of pan genes. (**b**) Rarefaction curve for the reduction of core genes. (**c**) Summary statistics for the 20,482 pan genes of *Halorubrum*. Cloud genes, 0% ≤ strains < 15%; shell genes, 15% ≤ strains < 95%; strict core genes, strains = 100%; soft core genes, 95% ≤ strains < 99%. The curves were fitted to median values of 1,000 permutations. The dark lines in (**a**) and (**b**) indicate median values, and the ‘shadows’ indicate the 95% confidence intervals. α = 0.564 ± 0.004 in (**a**) and α = 0.201 ± 0.011 in (**b**), indicating an open pangenome and core genome of *Halorubrum*. (**d**) Functional distribution of core genes (strict core genes plus soft core genes), shell genes and cloud genes for *Halorubrum*

The 70 *Halorubrum* genomes constituted an open pangenome with alpha value = 0.564 ± 0.004 (an alpha value < 1 is considered to indicate an ‘open’ pangenome, Fig. 2a) [30]. From the curves in Fig. 2a and b, it was predicted that about 130 more genes will be found once a new genome is added to the pangenome, and about three core genes will be excluded. The 70 genomes under study here were found to share 1,215 core genes (Fig. 2b). Of the 20,482 genes in the pangenome, most (78.63%) were present in < 15% strains, representing the cloud genes (Fig. 2c). The shell genes (present in 15% ≤ isolates < 95%) made up 13.09% of the pangenome, and the remaining 8.28% were therefore identified as core genes (both strict and soft core genes, present in ≥ 95% strains, Fig. 2c).

Based on the COG system, the core genes (both strict and soft core genes), shell genes and cloud genes of *Halorubrum* could be assigned to an equal number of nineteen functional categories (Fig. 2d). The proportion of genes assigned to each of the following categories showed a downward trend from core genes to shell and cloud genes: ‘energy production and conversion’, ‘cell cycle control, cell division, chromosome partitioning’, ‘coenzyme transport and metabolism’, ‘translation, ribosomal structure and biogenesis’, ‘posttranslational modification, protein turnover, chaperones’ and ‘intracellular trafficking, secretion, and vesicular transport’ (Fig. 2d). By contrast, functional categories related to ‘cell wall membrane envelope biogenesis’, ‘signal transduction mechanisms’, and ‘defence mechanisms’ accounted for increasing proportions from core genes to shell and cloud genes (Fig. 2d). About 31%, 43% and 42% of the core, shell and cloud genes, respectively, could not be assigned to categories with a known function (Fig. 2d).

### Overview of the *Halorubrum* genomes

**Fig. 3:**
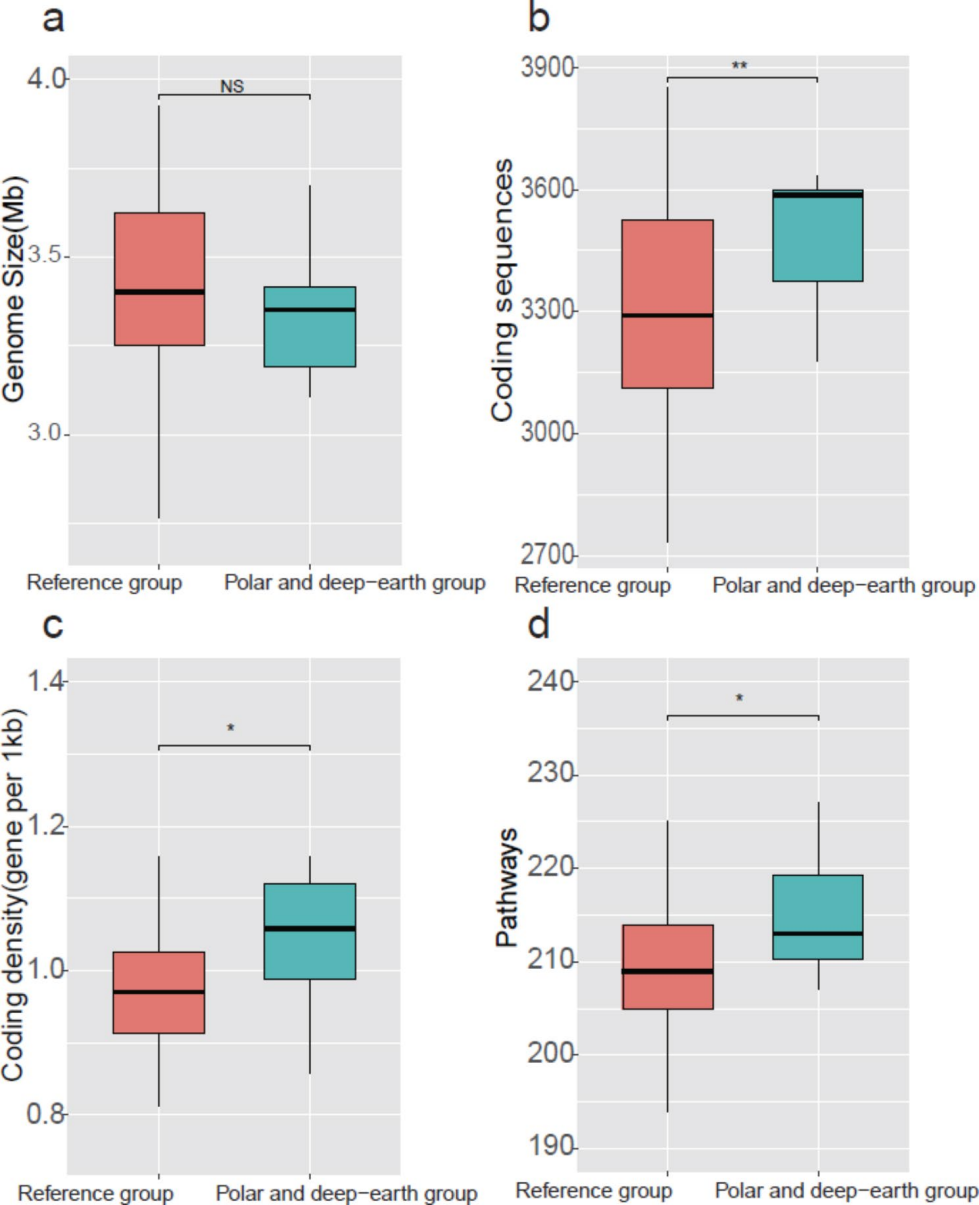
Comparison of the (**a**) genome size, (**b**) number of coding sequences, (**c**) coding density and (**d**) number of complete metabolic pathways between the polar and deep-earth group and the reference group. The polar and deep-earth group had a significantly higher number of coding sequences, higher coding density and more complete metabolic pathways than the reference group; no significant difference in genome size was found (Wilcoxon test, *, *p* < 0.05; **, *p* < 0.01; NS, not significant)

The *Halorubrum* genomes ranged in size from 2.77 Mb (*Halorubrum* sp. C191, isolated from an endorheic salt lake) to 3.93 Mb (*Halorubrum* sp. GN11GM_10 − 3_MGM, isolated from saturated brine), with a mean value of 3.40 Mb ± 0.24 Mb (roughly equivalent to the sizes of *Hrr. lacusprofundi* HLS1 and *Hrr. lacusprofundi* DL18, isolated from Deep Lake). The genomic G + C content of *Halorubrum* ranged from 62.90% (*Hrr. vacuolatum* DSM 8800, isolated from a saline lake) to 69.10% (*Halorubrum* sp. ATCC 700,873, isolated from a salt mine), and averaged 67.26% ± 1.23%. A comparison of genome size between the PD group and the reference group showed that there was no significant difference (Wilcoxon test; *p* > 0.05; Fig. 3a). However, the PD group had significantly higher numbers of coding sequences and higher coding density, indicating denser packing of genes (Wilcoxon test; *p* < 0.05; Fig. 3b and c). The higher coding density resulted in more metabolic pathways, with averages of 212 and 209 metabolic pathways in the PD group and the reference group, respectively (Wilcoxon test; *p* < 0.05; Fig. 3d; Table S2). The majority of the metabolic pathways that were overrepresented in the PD group were related to glycolysis and gluconeogenesis, the central carbon metabolic pathways that generate energy and mediate the synthesis of biomolecules [31].

### Genome-wide and RNA G + C content of *Halorubrum*

**Fig. 4:**
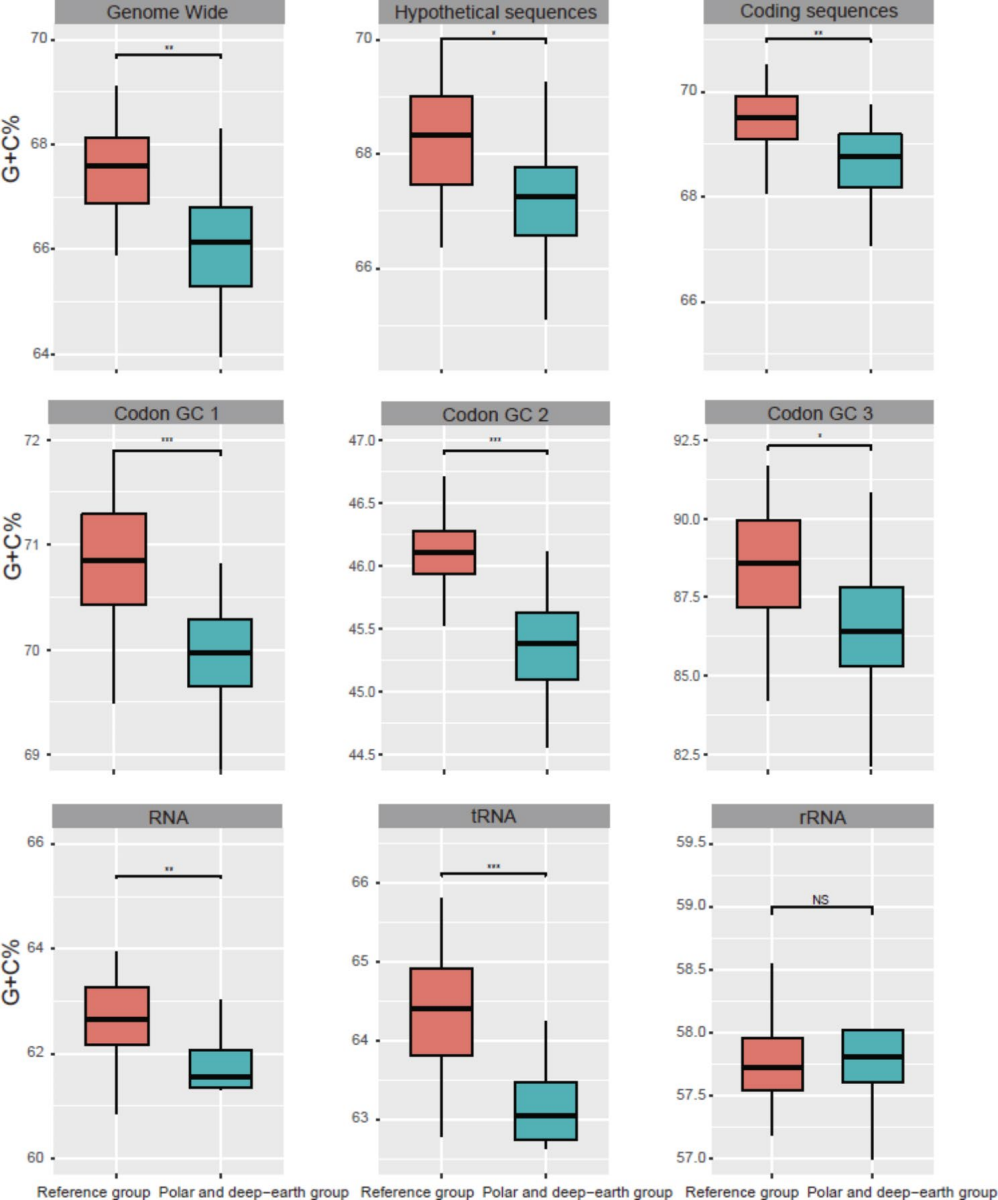
Comparison of the G + C content of genomic elements between the polar and deep-earth group and the reference group. Local G + C content of GC1, GC2 and GC3 (i.e. G + C composition at the first, second and third sites of genetic codons) (Wilcoxon test; *, *p* < 0.05; **, *p* < 0.01; ***, *p* < 0.005; NS, not significant; RNA including tRNA and rRNA)

The G + C content of genomic DNA, hypothetical sequences, and coding sequences was significantly lower in the PD group than the reference group (Wilcoxon test; *p* < 0.05; Fig. 4). We further calculated the G + C content at the three amino acid positions constituting a codon (GC1, GC2, GC3) for each of the coding sequences, and found that it was significantly lower at all three of the codon positions of the PD group’s coding sequences, compared to the reference group (Wilcoxon test; *p* < 0.05, Fig. 4). The decrease in G + C content could be detected when considering tRNA and rRNA as whole and tRNA only (Wilcoxon test; *p* < 0.05; Fig. 4), but it was not detectable for rRNA only (Wilcoxon test; *p* > 0.05; Fig. 4).

### Amino acid composition in *Halorubrum*

**Fig. 5:**
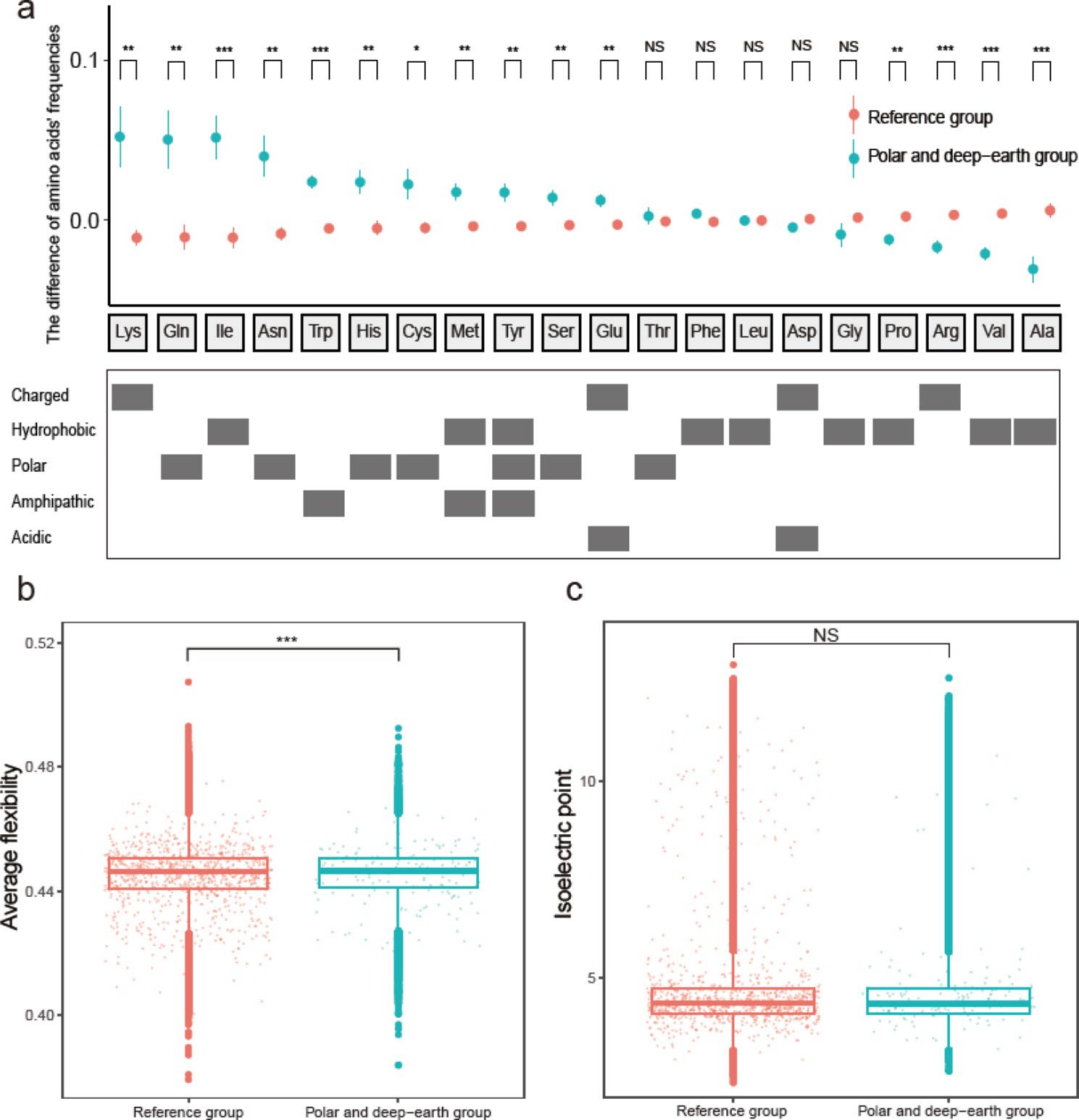
Optimization of genome-wide amino acid composition increased average flexibility but not the isoelectric point of the polar and deep-earth (PD) group *Halorubrum*. (**a**) Comparison of genome-wide amino acid composition of *Halorubrum*; the mean difference (colored circles) and standard deviation (vertical bars drawn through the circles) are plotted in the upper panel. *p-*values were obtained using the Wilcoxon test; the asterisks above the plot indicate significant differences between the polar and deep-earth group and the reference group. The lower panel shows the general chemical characteristics of each amino acid. (**b**, **c**) Comparison of protein flexibility (**b**) and protein isoelectric point (**c**) of the reference group and PD group *Halorubrum* at the genome scale. *, *p* < 0.05; **, *p* < 0.01; ***, *p* < 0.005; NS, not significant

Of the 20 standard amino acids, 11 were found to be present in significantly increased proportions in the PD group, relative to the reference group (lysine, glutamine, isoleucine, asparagine, tryptophane, histidine, cysteine, methionine, tyrosine, serine and glutamic acid) (Wilcoxon test; *p* < 0.05; Fig. 5a). Four amino acids (proline, arginine, valine and alanine) were found to be present in decreased proportions in the PD group (Wilcoxon test; *p* < 0.05, Fig. 5a). Two charged amino acids (Lys and Glu) were present in increased proportions in the PD group; one (Arg) was found to have decreased and one (aspartic acid) remained unchanged (Fig. 5a). Of the hydrophobic amino acids, three (Ile, Met and Tyr) were present in increased proportions and two (Pro, Val and Ala) in decreased proportions; three (phenylalanine, Leu and glycine) remained unchanged (Fig. 5a). All of the amphipathic amino acids (Trp, Met and Try) and the acidic amino acid Glu were found to be present in increased proportions in the PD group (Fig. 5a). This optimization of amino acid composition results in a significant increase in average flexibility, a good proxy of protein cold adaptation [17, 32] (Wilcoxon test; *p* < 0.05, Fig. 5b); however, it was not found to be associated with a significant change in isoelectric point between PD group and the reference group (Wilcoxon test; *p* > 0.05; Fig. 5c).

### Functional potential of *Halorubrum*

**Fig. 6:**
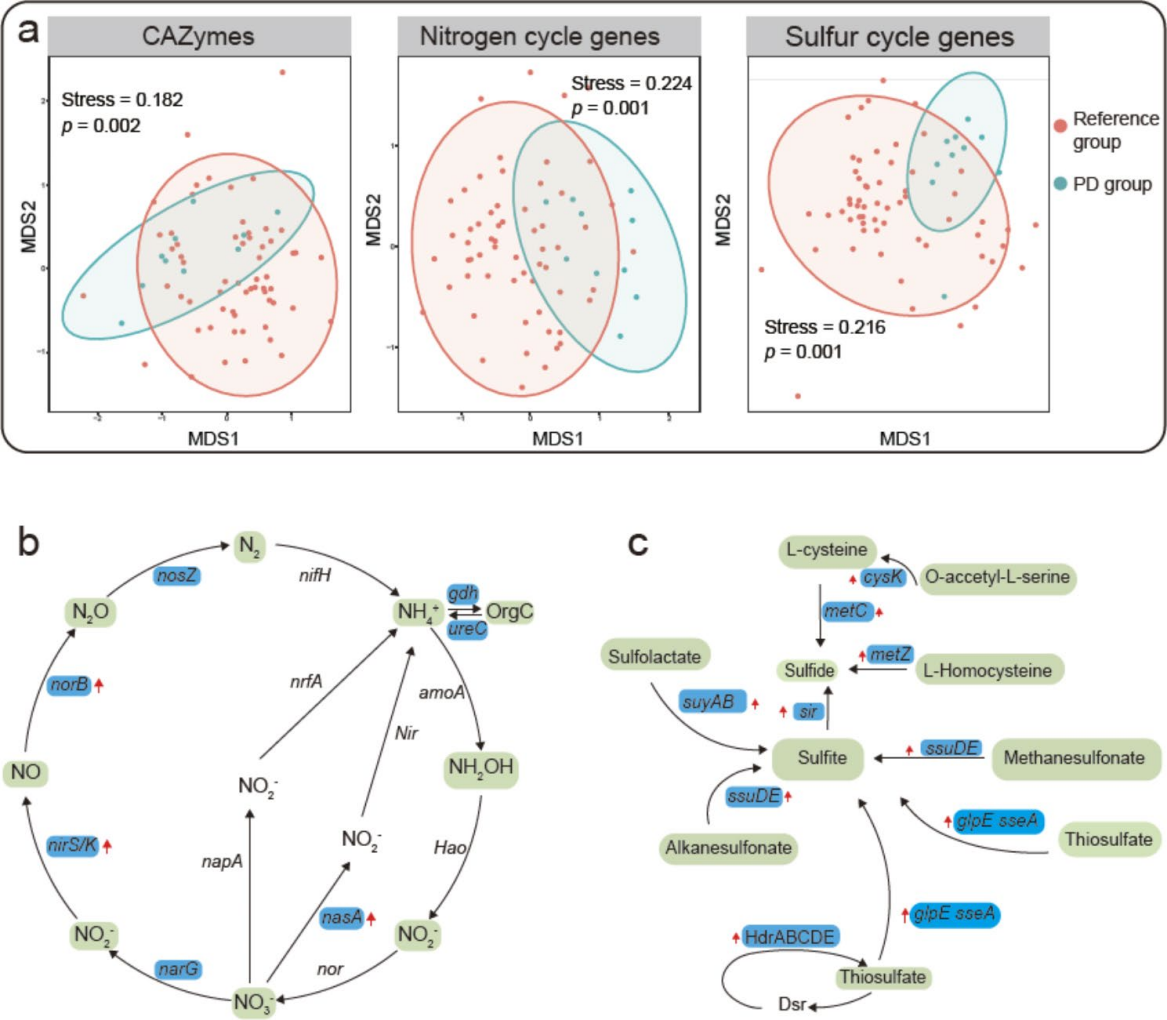
Comparison of functional capacity between the polar and deep-earth (PD) group *Halorubrum* and the reference group. (**a**) Nonmetric multidimensional scaling (NMDS) plots of CAZymes (carbohydrate-active enzymes) and nitrogen and sulfur cycling genes constructed based the Bray–Curtis dissimilarity. Analysis of the mean number of genes related to CAZymes, the nitrogen cycle and the sulfur cycle revealed a significant difference between the two groups (PERMANOVA; *p* < 0.05). The hulls indicate the 95% confidence intervals for a multivariate *t*-distribution for the respective groups of *Halorubrum*. (**b**) Summary of nitrogen cycling genes in *Halorubrum*; red up-arrows indicate genes that were enriched in the PD group. (**c**) The genes involved in organic and inorganic sulfur transformation pathways are shown in blue boxes; the red up-arrows denote the overrepresentation of these genes in the PD group *Halorubrum*. Modified from Yu et la., 2020. Note that the NMDS stress values of N and S cycling genes fall into the 0.2–0.3 category

The PD group was significantly different from the reference group in terms of gene content, specifically for genes related to CAZymes (carbohydrate-active enzymes), the nitrogen cycle and the sulfur cycle (PERMANOVA, *p* < 0.05, Fig. 6a). Analysis of the overall carbon cycle scheme indicated that *Halorubrum* are typical heterotrophic microorganisms which use organic carbon, including acetate and ethanol, as their main energy source (Fig. S2). For the carbohydrate-active enzymes, AA3_2 were depleted (Wilcoxon test; *p* < 0.05; Table S3). With regard to the nitrogen cycle, *Halorubrum* were predicted to be able to reduce NO_3_^–^ to N_2_ or NH_4_^+^; the nitrogen cycle genes identified as being significantly enriched in the PD group were *nirS/K* (for reducing NO_2_^–^ to NO), *norB* (for reducing NO to N_2_O) and *nasA* (for reducing NO_3_^–^ to NO_2_^–^) (Fig. 6b). The absence of *nifH* and *amoA* indicates that *Halorubrum* may not be able to fix nitrogen or oxidize ammonia (Fig. 6b).

The reconstruction of the sulfur cycling pathway showed that the cold-adapted *Halorubrum* species encoded most of the key genes for both organic and inorganic sulfur transformation. The majority of the enriched genes in the PD group were involved in the production of sulfite from organic-sulfur-containing compounds – for example, in the production of sulfite from methanesulfonate, thiosulfate, alkanesulfonate, and sulfolactate by *ssuDE, glpE, sseA, ssuDE* and *suyAB*; and sulfite could be further reduced to sulfide by *sir* (Fig. 6c). Genes *metC, metZ* and *cysK*, which are involved in the production of sulfide from L-homocysteine, O-accetyl-L-serine and L-cysteine, were found to be enriched in the PD group *Halorubrum* (Fig. 6c).

## Discussion

A body of studies has revealed that *Halorubrum* harbours diverse species, and there are members of *Halorubrum* that are well adapted to low temperatures and are abundant in cold saline lakes such as Deep Lake [11, 33]. *Halorubrum*-related sequences were also found to be abundant in the 2 m sample of the permafrost from the Canadian high Arctic [24]. Despite the competitiveness of *Halorubrum* in different cold environments globally, it is challenging to grow them at temperatures lower than 4 °C in the laboratory, and *Halorubrum* can grow at temperatures higher than 40 °C [2, 25]. Indeed, it is difficult to ascertain how well-adapted and ecologically important a microorganism is based on laboratory temperature-dependent growth curve tests [34]. Thus, there is a lack of knowledge regarding the genetic boundary between the cold-adapted *Halorubrum* species and their counterparts that thrive at higher temperatures.

In our analysis of the pangenome of *Halorubrum*, we found that the core genes exhibited a similar degree of functional diversity to the shell genes and cloud genes. This may confer on each member of *Halorubrum* the functional capacity to colonize a wide range of habitats. Having an open pangenome also indicates that *Halorubrum* is beginning to expand in terms of function and exploring new ecological niches [35]. There is an emerging view that the ecological theory developed for animals and plants may apply to Bacteria and Archaea [36]. Thus, the ability to occupy diverse and geographically distant habitats may be one of the reasons why *Halorubrum* is one of the largest haloarchaeal genera and has rapidly changing variation of its populations [37].

With respect to the high intrageneric diversity of *Halorubrum*, a clade containing 60% isolates from deep subterranean salt mines and Deep Lake was identified in the phylogenomic tree (i.e. the PD group). Although the ambient temperatures (~ 15 ℃) of the deep salt mines are not as low as those of Deep Lake (< 15 ℃ all year and < 0 ℃ for ~ eight months of the year), they can be considered low-temperature environments relative to saline lakes and solar salterns, where temperatures are around 20–30 ℃ [27, 33]. We hypothesize that this clade represents a low-temperature-adapted ecotype in *Halorubrum*. This is generally supported by the growth tests described in this study and in a previous study by Williams et al., (2017), both of which found that isolates from the PD group grew well at low temperatures and outperformed the reference isolates.

To further test our hypothesis, we then analysed the DNA G + C content of the PD group. For Bacteria and Archaea, the G + C content of genomic elements, especially RNAs, is a good proxy for temperature adaptation [38]. The lower G + C content of genomic DNA and RNA genes in the PD group, relative to the reference group, corresponds well to the improved ability of members of the PD group to survive at lower temperatures. The PD group is located in the middle of the phylogenomic tree shown in Fig. 1, and both its genomic and RNA G + C content are lower than those of the upper and lower clades, further supporting the notion that the lower G + C content is correlated with low-temperature adaptation rather than phylogenetic divergence.

The optimization of amino acid composition in the PD group provided stronger evidence that the PD group represents a low-temperature adapted ecotype in *Halorubrum*. In a comparison of the amino acid composition of the PD and reference groups, significant changes in the proportions of 15 of the 20 standard amino acids were observed. Significant decreases in the proportions of Arg and Pro, and significant increases in Lys and Asn, all of which represent well-established signatures of cold adaptation, were identified in the PD group [6, 39, 40]. By clearly delineating the cold-adapted ecotype of *Halorubrum*, we are now able to summarize, in statistical terms, the overall amino acid optimization of *Halorubrum* in response to low-temperature environments. We identified a bias in amino acid composition toward Lys, Gln, Ile, Asn, Trp, His, Cys, Met, Tyr, Ser and Glu and against Pro, Arg, Val and Ala. Our findings in the PD group were consistent with trends identified in psychrophilic *Arthrobacter*, a genus of bacteria in the Actinomycetes family; which reported similar findings for eight of the eleven amino for which an increase was observed (Asn, Lys, Met, Ile, Ser, Gln, Trp and His) and three of the four for which a decrease was observed (Ala, Pro, and Arg) [41].

The optimization of protein amino acid composition would enhance the activity of enzymes at low temperatures via a reduction in the number and strength of salt bridges (i.e. Asp-Arg salt bridge to Asp-Lys salt bridge) and would confer conformational flexibility and reduce activation energy [17, 42]. A comparison of the average flexibility between the PD group and the reference group further supported the idea that amino acid optimization has enabled genome-scale cold-environment adaptation in the PD group [6, 39]. The substitution of Lys for Arg may also help to reduce the amount of nitrogen needed for cell replication, as Lys has lower nitrogen content [43]. It is worth noting that the amphipathic amino acid content (for all three – Trp, Met and Try) was higher in the PD group; this indicates that the amphipathic amino acids may present a novel signature of cold adaptation that has not been noted in previous studies. We also observed that the optimization of amino acid composition by the PD group had not resulted in an increase in isoelectric point, which was thought to be incompatible with cold adaptation [44]. We speculate that the PD group *Halorubrum* are adapted genetically to the cold but that other unknown growth requirements prevent them from growing at temperatures < 4 °C.

Although it is clear that the optimization of amino acid composition to increase protein flexibility is a good indicator of cold adaptation in both archaea and bacteria, there is no general trend when classifying the amino acids based on their chemical characteristics only. For example, the hydrophobic amino acids Ile and Met were increased while Pro and Ala content were decreased in the cold-adapted clades of *Halorubrum* and *Arthrobacter*; Lys and Arg both have positive charges at neutral pH values but showed opposite trends in adapted proteins [41]. The trend of decreased Leu content identified in previous studies was not seen in the cold-adapted clades of *Halorubrum* or *Arthrobacter* [9, 41]; thus, based on statistical analyses of multiple closely related genome data sets, Leu may not be the key amino acid in low-temperature adaptation. The contrasting trends observed for some amino acids in different cold-adapted taxa probably result from a balancing of the overall amino acid composition.

The PD group was found to have higher functional potential with constant genome size relative to the reference group. This suggests that the PD group had higher substrate- and energy-use efficiency, enabling these species to drive the biogeochemical cycle in the oligotrophic cold polar and deep-earth environments. We further compared the functional traits between the PD group and the reference group by dividing genes into different functional categories. The PD group was shown to differ from the other *Halorubrum* in terms of overall gene content and specific functional genes involved in carbohydrate metabolism, the nitrogen cycle and the sulfur cycle. Functional differentiation between the PD group and the reference group further supports the idea that the PD group represents a low-temperature adapted ecotype in *Halorubrum*. The denser packing of genes indicates that the PD group may have undergone stronger positive selection of related genes [35]. We can explore the specific biogeochemical role of this cold-adapted clade using the reverse ecology principle, which states that the genome of an organism includes identifiable adaptational features to its native environment [36].

In our analysis of the nitrogen cycle, genomic data indicated that *Halorubrum* were able to reduce NO_3_^–^ to N_2_ or NH_4_^+^ but were not able to fix nitrogen or oxidize ammonia; this is consistent with the physiology of *Halorubrum* [45]. The genes *nirS/K*, *norB* and *nasA* were significantly enriched in the PD group, suggesting enhanced reduction of NO_3_^–^ by the cold-adapted *Halorubrum* species in polar and deep-earth hypersaline environments [45]. Our result is consistent with the findings that most of the genes involved in the denitrification pathway could be detected in Arctic permafrost, but the relative gene abundances for N_2_ production were low, leading to the accumulation of N_2_O, another greenhouse gas [46, 47].

The cold-adapted *Halorubrum* isolates encoded a number of key genes involved in both organic and inorganic sulfur transformation, and were especially enriched in genes involved in organic sulfur transformation. This suggests that the cold-adapted *Halorubrum* species prefer organic sulfur to generate energy for cellular activity and growth. Our results corroborated the findings of previous studies, in which strong psychrophilic adaptation of the sulfate reducers was identified in the Arctic sediment, and psychrophilic *Arthrobacter* were characterized as harbouring a complete mycothiol (MSH, a sulfur-containing compound) biosynthesis pathway [41, 48]. The capture of advantageous genes – such as those discussed here that confer on the PD group *Halorubrum* the ability to explore new ecological niches (i.e. deep subterranean salt mines and polar lakes) – can lead to the expansion of genomes. The fact that the genome sizes in the PD group *Halorubrum* remained constant suggests that the capture of new genes in this group might have overridden the selection for genome streamlining [35, 49]. This also implies that the genome content of the PD group *Halorubrum* is optimized such that maximum metabolic complexity is achieved without the cost of having increased the number of regulatory genes [50, 51].

Ordering genomes from geographically distant locations with similar low-temperature conditions into ecologically cohesive units helps to improve our understanding of the genomic features that are statistically associated with particular environmental conditions. However, it is difficult to identify a strict monophyletic group in which all isolates are from cold environments (e.g. polar, high alpine, and deep-earth environments) [41]. In this study, the PD group was found to harbour four isolates that were not from polar or deep-earth environments; however, these isolates formed a mixed clade with the polar and deep-earth isolates and shared conserved genomic traits. The benefits of defining the PD group were achieved at the expense of including the four non-cold-environment-derived isolates. However, there are in principle strict limits to what can be achieved by any simple system of classification; for example, in the classification of terrestrial climate, some locations may simultaneously satisfy the criteria for more than one category [52].

## Conclusions

By adding four isolates from deep salt mines to a clade anchored by the well-studied psychrophilic *Hrr. lacusprofundi* strains HLS1 and DL18, we have expanded the range of sources of cold-adapted *Halorubrum* species – which were previously limited to Antarctica – to include deep-earth environments. We also analysed the genomes of new PD group *Halorubrum* isolated from subterranean salt mines and reconstructed their C, N and S cycling capacities. In comparison to the reference group, the PD group *Halorubrum* possessed distinct genomic signatures consistent with their representation in low-temperature environments, and more compact genomes.

In the era of next-generation microbiology, two advances will improve our understanding of the genetic basis of environmental adaptation: (i) increasing the availability of multiple genomes sharing similar environmental conditions [21]; and (ii) minimizing the phylogenetic distance between target groups and reference groups [35, 53]. In this study, we defined a cold-adapted clade in *Halorubrum* harbouring 10 non-redundant genomes, for which phylogenetic noise was reduced as much as possible by using all other *Halorubrum* genomes as a reference group. Thus, an important step has been taken towards achieving the two advances that will improve our understanding of the environmental adaptation of microbes.

## Materials and methods

### Strain isolation and genome sequencing

Five haloarchaeal strains (*Halorubrum* sp. T3, Y78, Y69, ZC67, and F4) were isolated from salt rocks collected from depths of 300–700 m in salt mines in Yunnan, China, as described previously [25]. Strain *Halorubrum* sp. LN27 was isolated from a salt mine in Anhui, China, at a depth of 350 m, as described previously [54]. JCM168 medium was used to cultivate the isolates (https://jcm.brc.riken.jp/en/).

Genomic DNA was extracted from the isolates using a TIANamp Bacteria DNA Kit (Tiangen, Beijing), following the manufacturer’s instructions. Using genomic DNA, paired-end libraries with an insert size of 500 bp were constructed and sequenced using an Illumina HiSeq 2000 platform. Prior to *de novo* sequence assembly, low-quality reads were filtered out using Fastp with the default options [55]. Filtered sequencing reads were assembled using SPAdes v3.13.1 with the default options [56]. The assembled genome sequences were deposited in the DDBJ/ENA/GenBank database with the assembly ID provided in Table S1.

### Preparation of *Halorubrum* genomes for analysis

In September 2020, we retrieved all *Halorubrum* genome sequences from GenBank, obtaining 94 genomes. With the addition of the *Halorubrum* sp. F4 genome sequenced in this study and the five genomes sequenced by the authors in previous studies [54, 57], we obtained a total of 100 *Halorubrum* genomes. As the assignment of taxonomy in NCBI is relaxed, the taxonomy of the raw genomes was re-classified using the classify_wf workflow implemented in GTDB-Tk v1.4.0 with the database GTDB R95 prior to downstream analysis [58].

An organism’s genome contains all of its biological information; higher-quality genomes are therefore more informative. Thus, the 100 raw genomes were subjected to the following quality control and deduplication processes. QUAST v4.6.1 was used for contig and N50 calculations [59], and CheckM v1.0.7 was used for the genome quality estimation for each genome, using the default options [60]. Next, genomes with > 300 contigs, N50 < 20 kb, completeness < 95%, and contamination > 5% were removed. After quality filtering, we deduplicated the genomes by removing those with average amino acid identity (AAI) ≥ 99.5%. Genome quality filtering and deduplication were performed according to Parks et al., (2017) [61] and Shen et al., (2021) [41]. AAI values were calculated using CompareM with the default options (https://github.com/dparks1134/CompareM). A total of 70 genomes met the quality control requirements, including all six of the genomes contributed by the authors (Table S1).

### Phylogenomic and genomic analysis

The *Halorubrum* phylogenomic tree was constructed using PhyloPhlAn3 [62]. The phylogenomic tree can be drawn in multiple different but equivalent forms, so to obtain a relatively fixed phylogenetic topology, the phylogenomic tree was sorted with increasing node order using FigTree 1.4.4 (https://github.com/rambaut/figtree/releases). The annotation of the genes was standardized by annotating all genomes using PROKKA v1.14.5 [63]. Genome-scale reconstruction of metabolic pathways and analysis of the biogeochemical profiles were performed using gapseq v1.2 [64] and METABOLIC v4.0 [65]. Genome-scale calculation of protein flexibility and their isoelectric points were performed using ProtScale (https://web.expasy.org/protscale/) and ipc v1.0 [66] .The *Halorubrum* pangenome was constructed using PEPPAN v1.0.5 with the gff files produced by PROKKA used as input; the result produced by the main program of PEPPAN was parsed using PEPPAN_parser with the arguments -t -c -a 95 [67]. Rarefaction curves of the pan- and core-gene numbers were visualized with a custom-made R script [68]. The assignment of COGs (Cluster of Orthologous Groups of proteins) was performed with eggNOG-mapper v2.1.9 (http://eggnog-mapper.embl.de/). In addition to the general functional annotations, we used more specific tools for an in-depth exploration of the carbon, nitrogen and sulfur metabolism potential of *Halorubrum*. Carbohydrate-active enzymes were predicted using dbCAN2 [69]. Genes involved in nitrogen and sulfur cycling were predicted using NCycDB and SCycDB and the accompanying scripts [70, 71]. All parameters were set as default for the genomic tools and scripts used above, except where noted. R v4.2.1 and ggplot2 v3.4.2 were used for statistical analysis and plotting [68, 72]. For mapping strain isolation sites, we used the following R packages: pacman v0.5.1, leaflet v2.1.2, ggmap v3.0.2, sp v1.6-0, maptools v1.1-6, maps v3.4.1 and tidyverse v2.0.0. Additionally, we used ggalluvial v0.12.5 and ggsci v3.0.0 to show the functional distribution of core genes, cloud genes, and shell genes in *Halorubrum*. We used R package ggsignif v0.6.4 to add significance markers. For comparing the amino genome-wide acid composition of the of *Halorubrum*, we used R packages reshape2 v0.9.3 and ggpubr v0.6.0. We also used R packages vegan v2.6-4 and dplyr v1.1.2 to calculate PERMANOVA and NMDS. *p*-values were adjusted for multiple testing where required with Benjamini-Hochberg [73].

### Electronic supplementary material

Below is the link to the electronic supplementary material.

Supplementary Material 1

Supplementary Material 2

Supplementary Material 3

Supplementary Material 4

## Data Availability

The genome sequences of strains T3, Y78, Y69, ZC67, and F4 have been deposited at GenBank under the accessions GCA_000296615, GCA_007671725, GCA_007671685, GCA_004114995 and JAPDFS000000000, respectively.
