# Supplementary material for: Genomic analyses reveal a low-temperature adapted clade in Halorubrum, a widespread haloarchaeon across global hypersaline environments
Source: BMC Genomics. 2023 Aug 31;24:508. doi: 10.1186/s12864-023-09597-7 (PMC10468875; doi:10.1186/s12864-023-09597-7)
Supplement: Supplementary file 4 — Supplementary Material 4 [file 12864_2023_9597_MOESM4_ESM.docx]

Genomic analyses reveal a low-temperature adapted clade in *Halorubrum*, a widespread haloarchaeon across global hypersaline environments

Liangzhong Chen^1,2,3^, Tao Hong^1^, Zirui Wu^1^, Weizhi Song^4^, Shaoxing Chen^1*^, Yongqin Liu^5^, Liang Shen^1,2,6*^

^1^College of Life Sciences, Anhui Normal University, Wuhu 241000, China

^2^Anhui Provincial Key Laboratory of Conservation and Exploitation of Biological Resources, Wuhu 241000, China

^3^Engineering Research Centre for Molecular Detection and Diagnostics Anhui, Anhui Normal University, Wuhu 241000, China

^4^Centre for Marine Bio-Innovation, University of New South Wales, Sydney, NSW 2052, Australia

^5^Center for the Pan-third Pole Environment, Lanzhou University, Lanzhou 730000, China

^6^State Key Laboratory of Tibetan Plateau Earth System Science, Environment and Resources (TPESER), Institute of Tibetan Plateau Research, Chinese Academy of Sciences, Beijing 100085, China

*Corresponding author.

E-mail addresses: shenliang@ahnu.edu.cn (L. Shen), chensx@ahnu.edu.cn (S.X. Chen).

Fig. S1 Comparison of growth profiles of the representative isolates from the polar and deep-earth group (LN27) and the reference group (T3) at 4 ℃.

Fig. S2 Summary scheme of carbon cycling processes of *Halorubrum*. Each arrow represents a single transformation to step within a cycle, arrows in red indicate at least one isolate was positive for the reactions. Carbon cycle scheme showing that *Halorubrum* are typical heterotrophic bacteria characterized as able to utilize organic carbon, ferment ethanol and oxidize acetate.


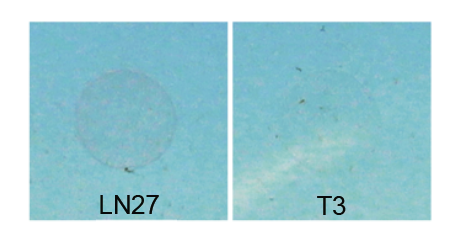


Fig. S1 Comparison of growth profiles of the representative isolates from the polar and deep-earth group (LN27) and the reference group (T3) at 4 ℃.


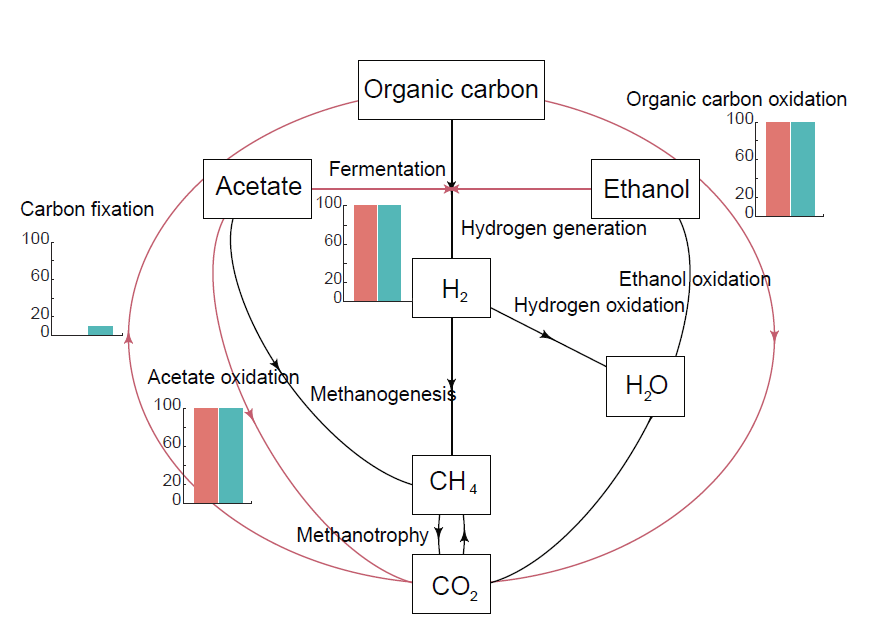


Fig. S2 Summary scheme of carbon cycling processes of *Halorubrum*. Each arrow represents a single transformation to step within a cycle, arrows in red indicate at least one isolate was positive for the reactions. Carbon cycle scheme showing that *Halorubrum* are typical heterotrophic bacteria characterized as able to utilize organic carbon, ferment ethanol and oxidize acetate.
